# Maternal Organ Growth: How the Adult Intestine Remodels During Pregnancy and Lactation

**DOI:** 10.1111/dgd.70037

**Published:** 2025-12-22

**Authors:** Tomotsune Ameku

**Affiliations:** ^1^ Institute of Science Tokyo, Institute of Integrated Research, Medical Research Laboratory Tokyo Japan

**Keywords:** intestinal epithelium, lactation, organ remodeling, pregnancy, tissue plasticity

## Abstract

Adult organs exhibit remarkable plasticity, dynamically adjusting their size and function to meet physiological demands. The small‐intestinal epithelium, one of the most rapidly renewing tissues in mammals, undergoes extensive growth and remodeling in response to diet, injury, microbiota changes, and reproduction. Reproduction is an energetically demanding process that requires precise regulation of maternal physiology to support fetal development and neonatal growth. In many mammals including humans, pregnancy induces systemic changes in hormones, metabolism, and immunity. At the organ level, pregnant and lactating females show increases in intestinal size across species such as mice, rats, sheep, and pigs—a phenomenon first documented nearly a century ago. However, the molecular mechanisms governing maternal intestinal remodeling during reproduction, and its physiological significance, remained unclear until recently. Emerging studies, including our recent work, have begun to reveal the cellular changes, molecular mechanisms, and triggers underlying this adaptive growth. This review summarizes current knowledge of intestinal epithelial plasticity in the context of reproduction, integrating findings from both reproductive and non‐reproductive settings. Understanding how the adult intestine adapts to physiological challenges offers valuable insights into developmental biology and has important implications for maternal metabolic health.

## 1 Introduction

Tissue plasticity in adult organs enables dynamic adjustment of organ size and function to meet physiological demands and environmental cues. The adult intestine undergoes marked remodeling in response to dietary interventions, microbiota depletion, tissue injury, and reproduction (Barker 2014; O'Brien 2022; Stojanović et al. 2022; Villablanca et al. 2022). Among these, reproductive growth is especially a physiologically relevant context: during pregnancy and lactation, the maternal small intestine grows, which is thought to support increased nutritional requirements. Nearly a century ago, Poo et al. (1939) reported increased weight and protein content in maternal organs, including the gastrointestinal tract, of pregnant and lactating rats. Subsequent rodent studies consistently demonstrated tissue‐level growth of the maternal intestine during reproduction (Boyne et al. 1966; Burdett and Reek 1979; Campbell and Fell 1964; Cañas et al. 1982; Craft 1970; Cripps and Williams 1975; Datta et al. 1995; Fell et al. 1963; Hammond et al. 1994; Harmatz et al. 1993; Prieto et al. 1994; Speakman and McQueenie 1996).

**FIGURE 1:**
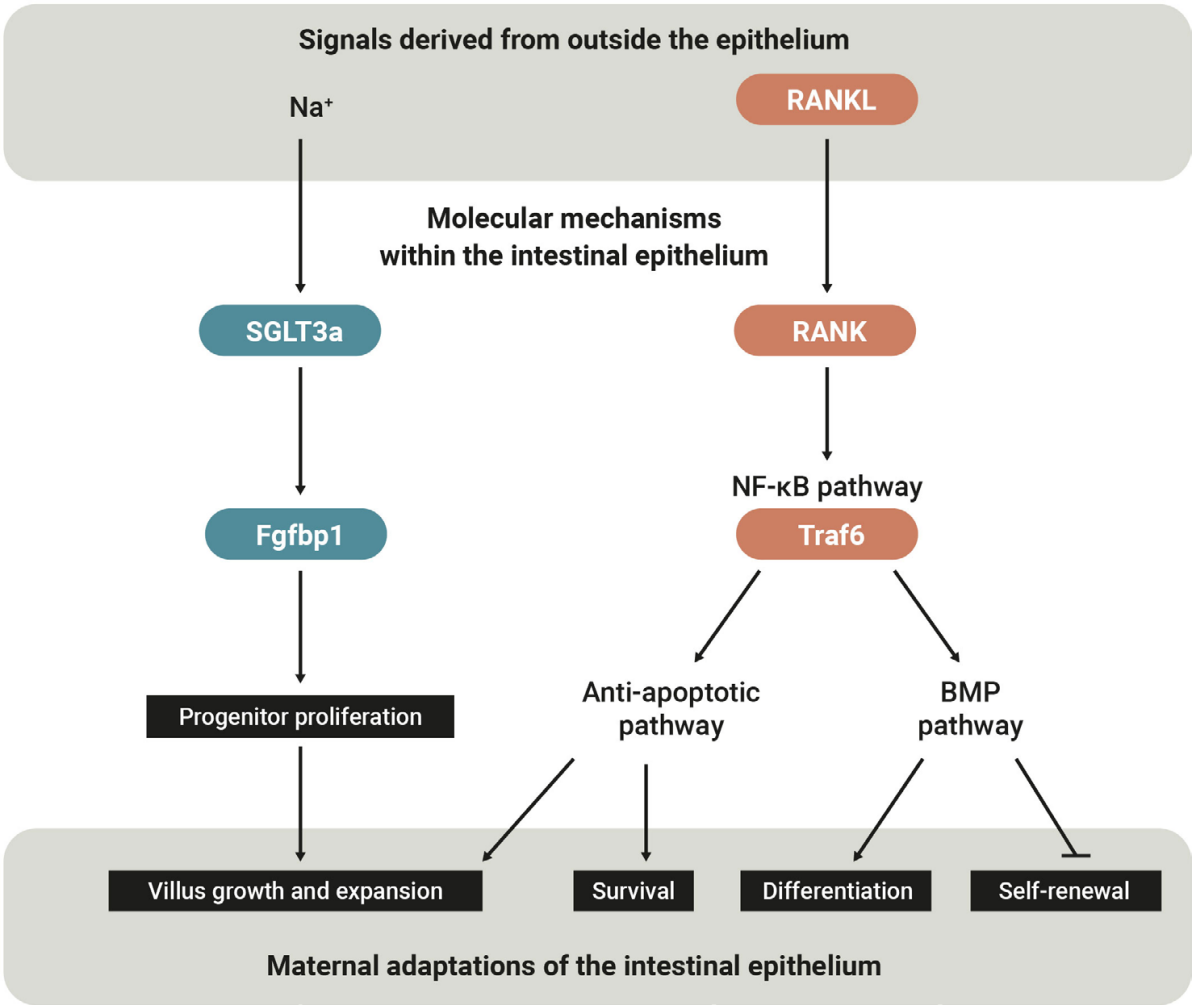
Molecular mechanisms regulating maternal intestinal epithelial growth. Two complementary molecular mechanisms regulating maternal intestinal epithelial growth have been described. Left: During pregnancy and lactation, the sodium‐ and proton‐sensitive transporter *SGLT3a* is upregulated in a subset of villus enterocytes. Rather than mediating digestion, SGLT3a non‐cell‐autonomously supports the expansion of adjacent *Fgfbp1*‐positive isthmus progenitors, thereby promoting villus growth. Dietary sodium supplementation in vivo can recapitulate villus expansion—supporting the functional relevance of this pathway for intestinal remodeling in the maternal state (Ameku et al. 2025). Right: Mesenchyme‐derived RANKL binds to RANK expressed in the intestinal epithelium, driving villus growth and expansion. The RANK‐RANKL interaction triggers NF‐κB signaling, which leads to the induction of anti‐apoptotic pathways and activates BMP signaling. This promotes the differentiation of intestinal epithelial cells while restricting stem cell self‐renewal (Onji et al. 2025). The interaction between SGLT3a and the RANK–RANKL pathway is currently unknown.

Work in *Drosophila* has identified molecular pathways underlying reproductive plasticity and sexual dimorphism in the adult digestive tract (Ahmed et al. 2020; Ameku et al. 2018; Blackie et al. 2024; Hadjieconomou et al. 2020; Hudry et al. 2016, 2019; Mineo et al. 2024; Reiff et al. 2015; White et al. 2021; Zipper et al. 2020). For example, Reiff et al. (2015) showed that the adult female midgut remodels after mating, driven by feedforward endocrine signaling that meets the increased metabolic demands of reproduction. In mammals, the molecular mechanisms underlying the growth of the maternal gut during reproduction remained unclear until recently; understanding these mechanisms is crucial for revealing how maternal organs adapt to the substantial physiological demands of pregnancy and lactation. Recent studies, including our own, have begun to elucidate the cellular changes, molecular mechanisms, and triggers involved in maternal gut remodeling (Ameku et al. 2025; Onji et al. 2025). Notably, these works describe pathways that may be complementary and together reveal the mechanisms by which reproductive growth of the intestinal epithelium is triggered (Figure 1).

Because maternal physiological changes are complex and occur across multiple levels, a central conceptual question is how mothers sense both internal physiological changes and external environmental cues and integrate these signals to orchestrate maternal organ remodeling during reproduction. Here, I review intestinal epithelial plasticity in the context of reproductive growth, integrating our recent findings with studies in non‐reproductive contexts to highlight shared and distinct mechanisms. This synthesis provides a framework for understanding how maternal physiology adapts to reproductive and metabolic demands through organ remodeling. It also offers insight into developmental biology, as well as maternal metabolic health and disease during pregnancy.

## 2 Maternal Organ Growth in the Small Intestine During Reproduction

### 2.1 Distinct Mechanisms Regulating Maternal Gut Elongation and Epithelial Growth

**FIGURE 2:**
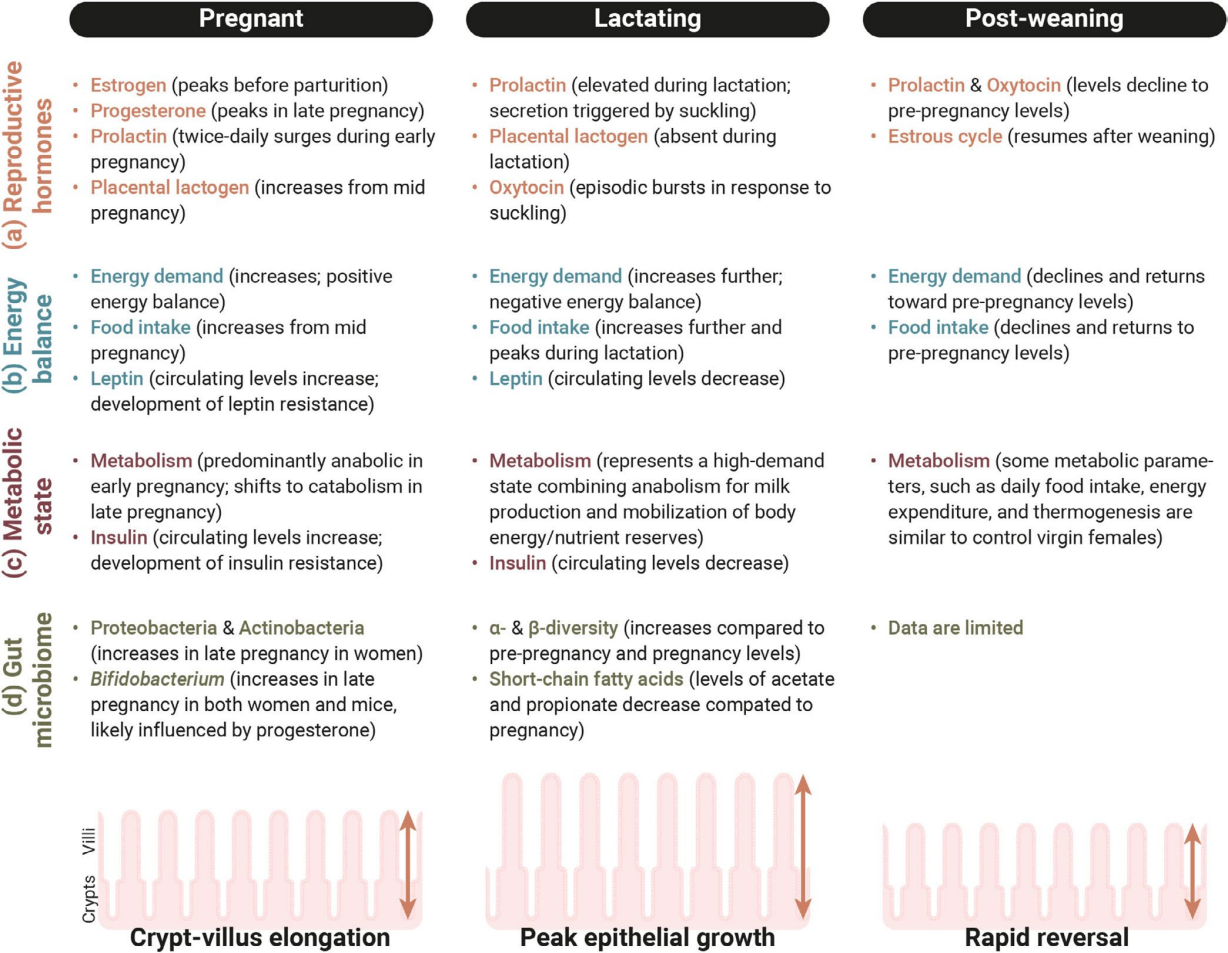
Overview of maternal changes in hormonal, metabolic, and gut microbial systems across reproductive stages. (a) Reproductive hormones: Several reproductive hormones show temporal changes during pregnancy and lactation (Georgescu 2023; Napso et al. 2018; Phillipps et al. 2020). Steroid hormones, such as estrogen and progesterone, gradually rise during pregnancy, peaking in mid‐ to late pregnancy and decline during lactation. In rodents, prolactin secretion is characterized by twice‐daily surges during early pregnancy, while placental lactogen begins to rise in early to mid‐pregnancy and continues to increase until parturition. Following a suppression of prolactin during mid‐pregnancy by placental lactogen, prolactin levels rise again in late pregnancy and remain high throughout lactation. During lactation, prolactin and oxytocin secretion are stimulated by suckling, and both hormones return toward pre‐pregnancy levels after weaning. (b) Energy balance: Pregnancy and lactation are energetically demanding states. In pregnant mice, energy demand increases not only to support the growing fetus but also to store energy for the future metabolic demands of lactation. This state of positive energy balance is characterized by increased maternal food intake (Hammond 1997; Speakman 2008) and leptin resistance, even as circulating leptin levels rise (Clarke et al. 2021; Ladyman et al. 2012; Li et al. 2021). In rodents, lactating females are typically in a state of negative energy balance, as their food intake, although markedly increased and peaking during lactation, is still insufficient to fully meet the energy requirements for milk production (Woodside 2007). Following weaning, energy demand gradually declines, and food intake decreases toward baseline (pre‐pregnancy) levels, although body weight may remain slightly elevated (Ladyman et al. 2018). (c) Metabolic state: In pregnant mice, metabolism is predominantly anabolic during early pregnancy, and shifts toward catabolic in late pregnancy to provide energy for the growing fetus. Circulating insulin levels increase during pregnancy, along with the development of insulin resistance (Khant Aung et al. 2020; Ladyman and Brooks 2021; Ladyman and Grattan 2017). Lactation represents a high‐demand metabolic state combining increased energy intake, anabolic processes for synthesizing milk components, and mobilization of body energy/nutrient reserves (Anhê and Bordin 2022; Hammond 1997; Yang et al. 2025). After weaning, some metabolic parameters, such as daily food intake, energy expenditure, and thermogenesis, are similar to control virgin females, but body weight remains elevated, suggesting a partial return to baseline energy homeostasis (Ladyman et al. 2018). (d) Gut microbiome: Maternal gut microbiota composition dramatically changes during pregnancy (Koren et al. 2024; Nuriel‐Ohayon et al. 2016). Phyla such as Proteobacteria and Actinobacteria increase from the first to the third trimester in women, and notably, transferring third‐trimester gut microbiota to germ‐free mice induces weight gain, insulin resistance, and a greater inflammatory response (Koren et al. 2012). The genus *Bifidobacterium* increases in both women and mice, likely influenced by progesterone (Nuriel‐Ohayon et al. 2019). In lactating mice, gut microbial alpha diversity (individual richness) and beta diversity (between‐subject diversity) increase, while levels of short‐chain fatty acids, particularly acetate and propionate, decrease compared with pregnancy (Guo et al. 2022). Data on the gut microbiota of post‐weaning dams are currently limited.

Maternal organ growth in the small intestine can be divided into two main aspects. One is an increase in tissue size—particularly focusing on elongation in this review—and the other is an increase in the surface area of the intestinal epithelium. These two processes are both observed as early as pregnancy day 7 and peak during lactation, suggesting that they share common temporal dynamics (Ameku et al. 2025; Hammond 1997; Onji et al. 2025; Speakman 2008). However, their regulatory mechanisms appear to differ. For example, in terms of reversibility, intestinal length remains significantly longer than that of virgin females 1 month (or even longer, about 200 days) after weaning (Ameku et al. 2025; Casirola and Ferraris 2003). In contrast, changes in the mucosal morphology (i.e., increases in villus height and crypt depth) are reversible—they return to pre‐pregnancy levels within 1 week after weaning/pup removal (Ameku et al. 2025; Onji et al. 2025). Consistent with these findings, intestinal length continues to increase cumulatively during a second lactation period, while villus height does not increase further after the first lactation (Ameku et al. 2025). These observations suggest that intestinal elongation and intestinal epithelial growth are regulated by distinct mechanisms during pregnancy and lactation, with intestinal epithelial remodeling (but not intestinal elongation) being tightly and dynamically regulated by the reproductive state. This distinction may reflect the need to balance long‐term structural adaptation with short‐term functional flexibility, allowing the maternal intestine to efficiently respond to both repeated reproductive demands and transient physiological states (Figure 2a–d). Given that multiple pregnancies are generally associated with larger litter sizes, the irreversible elongation of the intestine might be an adaptive change.

Although the underlying mechanisms may differ, organ‐level intestinal elongation is likely linked to the regulation of mucosal architecture, as the number of villi and crypts should increase proportionally when the intestine elongates—unless their density decreases, which we did not observe. Crypt fission, a process in which a single crypt bifurcates into two daughter crypts, has been implicated in intestinal growth during neonatal development and in regenerative contexts (Cairnie and Millen 1975; Cheng et al. 1986; Dudhwala et al. 2020). This suggests that crypt fission may serve as a reserve mechanism for expanding the epithelial surface in response to physiological demands such as reproduction. It may therefore be worth investigating whether maternal intestinal elongation is driven, at least in part, by epithelial remodeling processes like crypt fission, which are rare under steady‐state conditions in adults (Bruens et al. 2017).

One important difference between these two aspects (epithelial remodeling vs. intestinal elongation) is that epithelial growth—reflected in mucosal architecture such as villi and crypts—occurs within the intestinal epithelium itself, while intestinal elongation involves changes in other tissue components; these may include immune cells, muscle layers, and enteric neurons. This raises the intriguing possibility that the maternal small intestine may undergo coordinated remodeling across both epithelial and non‐epithelial tissues. Such interactions could play a critical role in supporting epithelial expansion—or, conversely, be driven by it. Investigating this crosstalk between epithelial and non‐epithelial components may uncover novel mechanisms by which the maternal intestine adapts structurally and functionally to the complex demands of reproduction.

### 2.2 Impact of Food Intake and Gut Microbiota on Maternal Gut Growth

A change in intestinal size is not limited during reproduction. For instance, food intake is a positive determinant of intestinal size: increased food intake results in increased intestinal size and surface area. Stojanović et al. (2021) investigated the relationship between the amount of food and intestinal size in a variety of mice which differed in the amount of eaten food. Excessive food intake in overeating mice who are genetically obese (mutants for satiety signals, leptin ligands, or its receptor), upon cold exposure and fed a low caloric density diet, results in increased small intestine length (Stojanović et al. 2021). Conversely, caloric restriction (restricted to 60% of the ad libitum standard diet) in wild‐type mice leads to a decrease in small intestine length and suppresses the elongation observed in overeating mice, as described above (Stojanović et al. 2021). Importantly, the positive effects of food amount on the small intestine also extend to absorptive villus height in this context. This observation raises a fundamental question about causality: is the maternal intestinal growth a passive response to increased nutritional load, or is it actively triggered by reproductive programs in anticipation of increased demands during reproduction? Indeed, maternal food intake dramatically increases during pregnancy and lactation (Alcantara et al. 2025; Hammond 1997; Speakman 2008), which is required for maternal gut growth to some extent (Datta et al. 1995). In our assays—both manual and automated measurement of daily food intake—we observe a significant increase in maternal food intake in mid‐pregnancy (pregnancy day 12 or 13, depending on the type of assay). On the other hand, maternal gut growth happens prior to this—we observe growth in villus height and crypt depth as early as pregnancy day 7 (Ameku et al. 2025), suggesting that additional factors may contribute to the initiation of maternal gut growth during early pregnancy. This early onset suggests that pregnancy‐associated hormonal changes may initiate maternal gut remodeling prior to increased food intake (Figure 2a,b). Such early intestinal adaptations may represent a feedforward mechanism preparing the maternal body for later nutritional demands.

In pregnant women, the composition of the gut microbiota changes dramatically from the first to the third trimester of pregnancy (Koren et al. 2012). When transferred to germ‐free mice, the third trimester microbiota induces metabolic changes in germ‐free recipient mice, suggesting the impacts of host–bacterial interaction on host metabolism during pregnancy (Koren et al. 2012). The question is whether the gut microbiota or its compositional changes are required for maternal gut growth during pregnancy. Indeed, the changes in gut microbiota impact the length of small intestine—a germ‐free treatment leads to a dramatic increase in their intestinal length (Al‐Asmakh and Zadjali 2015; Chevalier et al. 2015), which can be interpreted as a compensation mechanism because the gut microbiota normally helps in digesting and absorbing for the host. Our group and Onji et al. (2025) have confirmed that maternal small intestine growth still occurs in germ‐free mice, suggesting that the gut microbiota is not required for inducing maternal gut growth during pregnancy (Ameku et al. 2025). In parallel, we also observed that probiotic supplementation (*Lactiplantibacillus plantarum*, WJL strain) (Schwarzer et al. 2016, 2023; Storelli et al. 2011) during pregnancy and lactation does not prevent maternal gut growth (Ameku et al. 2025). These data indicate that even though pregnancy remodels the gut microbiota, which impacts the gut remodeling in the host independent of reproduction, maternal gut growth is not a result of the changes in gut microbial composition during pregnancy. Therefore, identifying microbe‐independent mechanisms—such as hormonal regulation—is essential to understand the initiation of maternal gut growth (Figure 2a,d).

### 2.3 Remodeling of the Intestinal Epithelium During Reproduction: Accelerated Turnover and Altered Cell Composition

The intestinal epithelium is among the most rapidly renewing mammalian tissues, maintained by coordinately regulated proliferation and differentiation. In the small intestine, stem cells in the crypts give rise to progeny that migrate into villi and differentiate into absorptive or secretory lineages (Cheng and Leblond 1974a, 1974b; Clevers 2013; Gehart and Clevers 2019; van der Flier and Clevers 2009). Proliferative *Lgr5*‐positive crypt base columnar (CBC) cells (Barker et al. 2007; Snippert et al. 2010) and *Fgfbp1*‐positive isthmus progenitors (formerly known as transit‐amplifying cells) (Capdevila et al. 2024; Malagola et al. 2024), located at the crypt base and upper crypt, respectively, generate multiple intestinal cell types. This includes secretory lineages—Paneth cells that produce antimicrobial peptides and niche signals, mucus‐secreting goblet cells, hormone‐producing enteroendocrine cells, and chemosensory tuft cells—and the absorptive lineage, represented by enterocytes for nutrient uptake.

**FIGURE 3:**
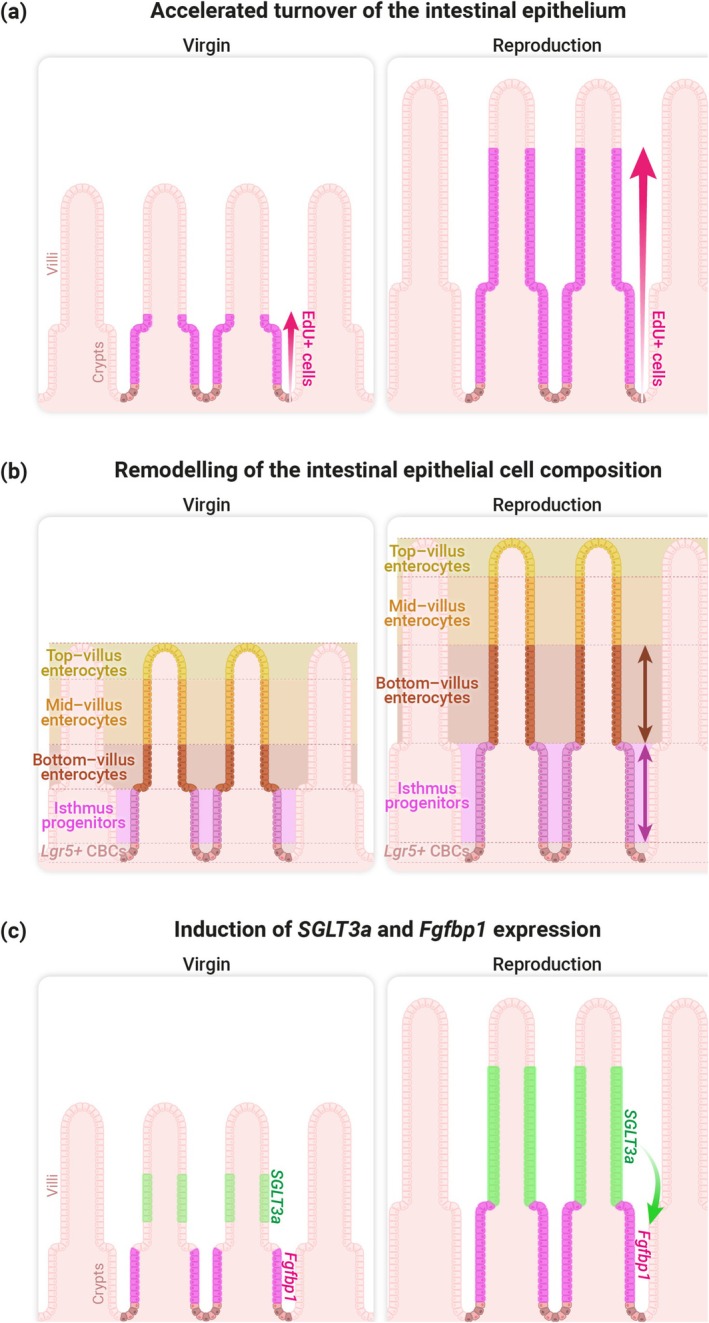
Maternal organ growth in the intestinal epithelium. (a) Accelerated turnover of the intestinal epithelium: During pregnancy and lactation, both the number of proliferating cells and the migration of differentiated cells increase, as shown by EdU pulse‐labeling and anti‐Ki67 immunostaining. EdU‐positive cells are shown in magenta (Ameku et al. 2025). (b) Remodeling of cell composition in the maternal intestinal epithelium: During reproduction, the crypt–villus unit not only enlarges in size but also undergoes remodeling of its cellular composition. In situ spatial imaging analysis reveals that, during lactation, the maternal intestinal epithelium preferentially expands in isthmus progenitors (shown in magenta) and bottom‐villus enterocytes (shown in brown), rather than increasing uniformly across all cell types (Ameku et al. 2025). (c) Reproductive induction of *SGLT3a* in enterocytes and *Fgfbp1* in isthmus progenitors: During pregnancy and lactation, the expression of *SGLT3a* (encoded by *Slc5a4a*) is significantly upregulated in the maternal intestinal epithelium. In situ spatial imaging, in situ hybridization analyses, and single‐cell RNA sequencing reveal that *SGLT3a* is highly expressed in bottom‐ and mid‐villus enterocytes (shown in green) but not in Ki67‐positive proliferative progenitors within the crypts. *Fgfbp1*‐positive isthmus progenitors expand during pregnancy and lactation (shown in magenta), a process mediated by *SGLT3a* upregulation in the villi through an unknown mechanism (Ameku et al. 2025).

The size of the crypt–villus unit is influenced by the number and/or size of epithelial cells. During pregnancy and lactation, both factors contribute to the elongation of the crypt–villus unit, as the intestinal epithelium exhibits increased cell size and enhanced proliferation activity (Ameku et al. 2025). The intestinal epithelium undergoes one of the most rapid turnover rates among all mammalian tissues, typically renewing every 3–5 days. This baseline rate is observed in virgin females, as demonstrated by EdU (5‐ethynyl‐2′‐deoxyuridine) incorporation experiments (Ameku et al. 2025). During reproduction, however, this turnover rate increases substantially, peaking during lactation when epithelial renewal occurs in just 1.3 days (Figure 3a) (Ameku et al. 2025). Importantly, this accelerated renewal is reversible: within 7 days after weaning, the turnover rate returns to baseline levels, reflecting the dynamic and reversible nature of crypt–villus remodeling (Ameku et al. 2025). As intestinal epithelial cells actively migrate up the villus via an actin‐mediated process after exiting the crypt—rather than passively moving as if on a conveyor belt (Krndija et al. 2019)—it would be interesting to investigate whether cytoskeleton remodeling occurs in the intestinal epithelium to accelerate epithelial turnover during reproduction.

The crypt–villus unit enlarges during reproduction, but does it also remodel in terms of cellular composition—and if so, how? Two hypotheses can be considered: (a) intestinal epithelial cell types expand proportionally, resulting in no change in overall composition, or (b) specific cell types expand preferentially, leading to a shift in cellular composition. To test these possibilities, we used in situ spatial imaging analysis to compare the composition of epithelial cell types—including *Lgr5*‐positive CBCs, isthmus progenitors, secretory cells, and absorptive enterocytes—between reproductive (pregnancy and lactation) and non‐reproductive (virgin) states. In lactating mice, we observed a significant increase preferentially in isthmus progenitors in the upper crypts, along with an expansion of adjacent bottom‐villus enterocytes, compared to virgin controls (Figure 3b) (Ameku et al. 2025). These changes may represent a cellular mechanism that supports the accelerated epithelial turnover observed during reproduction. To further investigate this shift in cellular composition, we performed in situ hybridization and found a significant increase in *Fgfbp1*‐positive isthmus progenitors during lactation, whereas *Lgr5*‐positive CBCs remained unchanged (Ameku et al. 2025). These findings support the conclusion that the proportion of isthmus progenitors increases within the epithelial cell population during reproduction. Taken together, these observations indicate that the maternal intestinal epithelium undergoes not only structural enlargement of the crypt–villus architecture but also remodeling of its cellular composition in response to the reproductive state. An important question is whether the intestinal stem cell niche—including epithelial cells, sub‐epithelial mesenchymal cells, lymphatic endothelial cells, and extracellular matrix components (Goto et al. 2022; McCarthy et al. 2020; Meran et al. 2017; Niec et al. 2022; Palikuqi et al. 2022)—is remodeled and how niche signals change to regulate intestinal stem cell behavior during reproduction, because Onji et al. (2025) have demonstrated that RANK–RANKL signaling drives maternal epithelial expansion and controls the intestinal stem cell niche through the BMP pathway (discussed in detail in Section 3).

Notably, we observed an increased proportion of enteroendocrine cells during pregnancy and an upregulation of genes encoding enteroendocrine hormones during lactation (Ameku et al. 2025). Among these, *Gcg* encodes proglucagon, a precursor that is post‐translationally processed into several peptide hormones—including glucagon‐like peptide‐1 (GLP‐1), glucagon‐like peptide‐2 (GLP‐2), and oxyntomodulin—depending on the tissue and the enzymes expressed (Drucker 1998). GLP‐2 is a plausible regulator of reproductive gut growth, as it promotes crypt cell proliferation and villus elongation under non‐reproductive conditions (Austin et al. 2016; Brubaker 2018; Drucker et al. 1996; Shin et al. 2005; Tsai et al. 1997).

*Lgr5*‐positive CBCs are central to intestinal epithelial renewal and the maintenance of homeostasis (Barker et al. 2007; Snippert et al. 2010). However, regeneration following damage—such as inflammation, irradiation, or genetic ablation—is supported by robust and redundant mechanisms within the crypts (Beumer and Clevers 2016). In response to crypt damage, several differentiated cell types—including enteroendocrine cell precursors (Buczacki et al. 2013; Yan et al. 2017), *Dll1*‐positive secretory progenitors (van Es et al. 2012), *Alpi*‐positive enterocyte progenitors (Tetteh et al. 2016), and Paneth cells (Yu et al. 2018)—can dedifferentiate and reacquire stem cell potential, thereby functioning as damage‐induced intestinal stem cells. In addition to these facultative sources, a population of quiescent “+4” cells—normally located at the +4 position relative to the crypt base—can become activated upon damage to help restore the *Lgr5*‐positive stem cell compartment (Higa et al. 2022; Li and Clevers 2010; Montgomery et al. 2011; Powell et al. 2012; Sangiorgi and Capecchi 2008; Takeda et al. 2011; Tian et al. 2011; Yan et al. 2012). Furthermore, *Sca1*‐positive cells (Nusse et al. 2018) and *Clu*‐positive revival stem cells (Ayyaz et al. 2019), which are absent under homeostatic conditions, emerge following crypt damage and contribute specifically to regeneration. This raises the question of whether maternal gut epithelial growth during reproduction is driven by mechanisms similar to those of damage‐induced regeneration to support accelerated turnover, a hypothesis that can be tested through lineage tracing experiments. Moreover, given the potential for dynamic mechanical remodeling of the maternal intestine during pregnancy and lactation (Mercado‐Perez and Beyder 2022; Mineo et al. 2024)—such as increased tissue tension and changes in basement membrane stiffness—piezo‐mediated mechanosensing (Baghdadi et al. 2024) may contribute to the regulation of intestinal stem cell proliferation and differentiation in response to these physiological cues during reproduction. Reproductive mechanical remodeling in the maternal intestine might also be associated with the principle that intestinal crypt formation is orchestrated by spatially organized mechanical forces and osmotic dynamics, which are coordinated through cell fate specification and tissue compartmentalization (Pérez‐González et al. 2021; Yang et al. 2021).

### 2.4 Metabolic and Physiological Adaptations of the Intestine During Reproduction

Maternal metabolism undergoes profound changes during reproduction (Haddad‐Tóvolli and Claret 2023; Yang et al. 2025; Yu et al. 2025). But how does reproductive state affect gene expression in the maternal intestine? Our transcriptomic analyses (Ameku et al. 2025) reveal the following: (1) Reproductive state has a greater impact on intestinal gene expression than sex, as shown by bulk RNA sequencing of whole intestinal tissues. Notably, genes involved in metabolism, signaling, and immune function are differentially expressed; (2) These transcriptional changes are particularly pronounced in absorptive enterocytes, where genes related to metabolic pathways—such as fatty acid and carbohydrate metabolism—and micronutrient absorption and transport are significantly altered, which are findings based on single‐cell RNA sequencing of FACS‐sorted intestinal epithelial cells during lactation. This transcriptional remodeling likely supports the increased metabolic demands of reproduction by enhancing nutrient absorption.

Independent of reproductive states, dietary modifications are widely recognized to influence intestinal plasticity (Stojanović et al. 2021, 2022): in both flies and mice, intestinal stem cells regulate gut growth and plasticity in response to dietary stimuli (Alonso and Yilmaz 2018; Novak et al. 2021; O'Brien et al. 2011; Shay and Yilmaz 2025). During caloric restriction, the number and proliferation of stem cells increase within the crypts (Igarashi and Guarente 2016; Yilmaz et al. 2012), while the villi become shorter and contain fewer enterocytes. This indicates that caloric restriction promotes intestinal stem cell self‐renewal at the expense of their differentiation (Yilmaz et al. 2012). Similar to caloric restriction, fasting is also known to induce stem cell proliferation in the crypts (Mihaylova et al. 2018; Richmond et al. 2015); importantly, short‐term refeeding after fasting further enhances proliferation and the regenerative capacity of intestinal stem cells by activating the mTORC1 pathway and promoting polyamine metabolism (Imada et al. 2024).

Furthermore, dietary composition plays a significant role in modulating stem cell activity; intestinal stem cell proliferation is enhanced by a high‐fat diet (Beyaz et al. 2016; Mana et al. 2021; Xie et al. 2020) and ketogenic diet (Cheng et al. 2019; Shay et al. 2025). Increasing cellular cholesterol—either by adding exogenous or dietary cholesterol or by upregulating cholesterol biosynthesis and uptake—has been associated with enhanced intestinal stem cell proliferation and consequently regeneration (Wang et al. 2018). Cellular and systemic cholesterol homeostasis is maintained by liver X receptor signaling, which plays a crucial role in uncoupling intestinal regeneration from tumorigenesis (Das et al. 2025).

A Western‐style diet high in both fat and sugar enhances intestinal stem cell proliferation and epithelial cell turnover accompanied by increased villus growth (Aliluev et al. 2021; Stojanović et al. 2021). Another Western‐style dietary component, namely high‐fructose corn syrup, causes intestinal villus elongation (Taylor et al. 2021). Interestingly, dietary fructose regulates villus growth by enhancing cell survival rather than cell proliferation (Taylor et al. 2021), which differs from the modulation of stem cell activity observed with high‐fat diet feeding (Stojanović et al. 2022). How diet‐driven regulatory mechanisms in the intestinal epithelium and stem cells contribute to maternal gut remodeling during reproduction remains unclear. However, it is possible that shared pathways underlie both dietary responses and reproductive gut adaptations. Exploring how diet and reproductive state interact will help us to better understand the processes that drive maternal gut growth and metabolic adaptation during reproduction.

Does reproductive state alter maternal intestinal physiology? Using ex vivo Ussing chambers, we found no significant differences in intestinal trans‐epithelial ion transport between virgin and lactating mice (Ameku et al. 2025). Surprisingly, digestive efficiency decreases rather than increases during lactation, as revealed by calorimetric analysis of fecal samples (Ameku et al. 2025). Thus, despite no apparent improvement in intestinal physiological efficiency during reproduction, total caloric intake increases—driven by increased food consumption—and overall trans‐epithelial ion transport would be elevated as a result of intestinal elongation. Transcriptomic metabolic changes, along with other physiological adaptations—such as reduced intestinal transit time (Harmatz et al. 1993)—may support reproductive nutritional demands by sustaining digestion and absorption along an elongated intestinal tract (Hammond 1997).

## 3 Molecular Mechanisms Driving Maternal Gut Growth

### 3.1 Extrinsic Signals Driving Maternal Epithelial Growth During Pregnancy and Lactation

What drives maternal gut growth at the molecular level—specifically, which driver gene initiates this growth rather than it being a consequence of gut remodeling? We and others have identified driver genes and molecular mechanisms that trigger maternal gut epithelial growth, potentially as a compensatory pathway (Figure 1).

Onji et al. (2025) demonstrated that signals originating outside the epithelium drive maternal intestinal epithelial expansion during pregnancy and lactation. This process is mediated by the receptor activator of nuclear factor κB (RANK), a transmembrane receptor, and its ligand RANKL, which are known to form a signaling pathway essential for bone remodeling (Kong et al. 1999; Simonet et al. 1997), immune regulation (Paolino et al. 2021), and mammary gland development (Fata et al. 2000). During pregnancy, the RANK–RANKL pathway also facilitates maternal intestinal adaptation to meet increased nutritional demands. In mice lacking this signaling, pregnancy‐induced villus elongation is impaired, leading to lower nutritional quality of milk, reduced offspring weight gain, and impaired maternal glucose tolerance under metabolic stress (Onji et al. 2025). Conversely, excessive RANK activation induces abnormal villus overgrowth, eventual depletion of *Lgr5*‐positive intestinal stem cells, and suppression of intestinal tumor formation (Onji et al. 2025).

Mechanistically, RANK–RANKL protects gut epithelial cells from apoptosis and modulates the intestinal stem cell niche through enhanced BMP signaling, thereby promoting villus elongation and expansion of the intestinal epithelium during reproduction. Consistent with these findings, RANKL expression tends to increase in gut lamina propria cells during lactation, with *Twist2*‐expressing mesenchymal cells and T cells serving as local sources. The ligand subsequently activates RANK signaling in intestinal epithelial cells to drive maternal villus expansion (Onji et al. 2025). Future work will be required to identify the upstream signals that regulate RANK–RANKL–mediated maternal intestinal adaptation, particularly prolactin, whose inhibition impairs maternal gut remodeling during lactation (Onji et al. 2025).

### 3.2 Driver Genes Are Upregulated Within the Intestinal Epithelium During Pregnancy and Lactation

We identified SGLT3a as the driver (Figure 1) and focused on it for the following reasons (Ameku et al. 2025): (1) Its gene expression is upregulated during lactation in our transcriptome datasets, including bulk RNA sequencing of whole intestinal tissues, bulk and single‐cell RNA sequencing of FACS‐sorted intestinal epithelium, as well as in situ spatial imaging analysis; (2) Its gene expression is also upregulated in early pregnancy (pregnancy day 7), a stage in which only 11 genes are differentially expressed compared to 90 genes during lactation. In our intersectional analysis, only two genes—*Slc5a4a* and *Slc31a1*—were upregulated in both early pregnancy and lactation.

*Slc5a4a* encodes SGLT3a, a member of the sodium‐glucose cotransporter (SGLT) family. In rodents, SGLT3 has two paralogs—*Slc5a4a* and *Slc5a4b*—which encode SGLT3a and SGLT3b, respectively. SGLT3 is a homolog of SGLT1, which mediates active glucose transport from the intestinal lumen into epithelial cells (Gorboulev et al. 2012). In contrast, the electrophysiological properties of SGLT3a differ from those of SGLT1 (Barcelona et al. 2012; Soták et al. 2017). Consistent with previous work (Barcelona et al. 2012), our electrophysiology experiments have demonstrated that SGLT3a is activated by protons but does not respond to sugars, including glucose, galactose, sucrose, arabinose, or fructose (Ameku et al. 2025). Notably, we found that SGLT3a is also activated by sodium, indicating a distinct electrophysiological property of SGLT3a compared with SGLT1 and SGLT3b.

Sodium emerged as an intriguing candidate in maternal gut growth during reproduction, as revealed by our dietary intervention experiment in vivo: a high‐sodium diet induced villus growth in virgin females, whereas no changes were observed in mice fed acidified water (Ameku et al. 2025). In mice fed a high‐sodium diet, *SGLT3a* expression was significantly decreased (unpublished data), suggesting that its expression may be regulated by dietary sodium availability or by the physiological demand for sodium. Further studies are required to determine whether dietary sodium is necessary for maternal gut growth during reproduction—for example, by assessing whether a low‐sodium diet impairs maternal gut growth during reproduction.

### 3.3 The Role of SGLT3a in the Regulation of Maternal Gut Epithelial Growth

To explore the in vivo functions of SGLT3a during reproduction, we first examined transgenic mice lacking both SGLT3a and SGLT3b proteins (hereafter referred to as SGLT3^KO^). We then validated relevant phenotypes and further investigated them using mutant mice lacking SGLT3a alone (hereafter SGLT3a^KO^). Our analysis showed that SGLT3a contributes to maternal gut epithelial growth: in SGLT3^KO^ mice, reproduction‐induced villus growth is reduced by 45% compared to wild‐type (WT) controls (Ameku et al. 2025). Notably, SGLT3 KO does not affect small intestine length or other organ sizes, regardless of reproductive status, indicating a specific role in gut epithelial growth (we confirmed that mutant females lacking SGLT3a alone exhibited phenotypes comparable to those of SGLT3^KO^ females). This effect is reproduction‐specific: villus height is unchanged by KO in virgin females, and high‐fructose‐induced villus growth (Taylor et al. 2021) occurs normally in SGLT3^KO^ mice, consistent with electrophysiological data showing SGLT3a does not respond to sugars, including fructose (Ameku et al. 2025).

How does reproductive induction of *SGLT3a* in enterocytes regulate villus growth? SGLT3a promotes accelerated epithelial turnover during reproduction, as shown by EdU incorporation and Ki67 staining in vivo (Ameku et al. 2025). Intestinal organoids derived from SGLT3a^KO^ mice exhibit reduced proliferation compared to WT controls, supporting a gut‐specific role in epithelial proliferation (Ameku et al. 2025). *SGLT3a* is expressed in absorptive enterocytes but not in Ki67‐positive proliferating cells, suggesting it regulates progenitor proliferation via a non‐cell‐autonomous mechanism.

During reproduction, the number of *Fgfbp1*‐positive cells increases, whereas the number of *Lgr5*‐positive CBCs remains unchanged (Figure 3c). SGLT3a is required for the expansion of *Fgfbp1*‐positive cells during lactation, as their numbers decrease in SGLT3a^KO^ mice (Figure 3c) (Ameku et al. 2025). Although cells co‐expressing *Slc5a4a* and *Fgfbp1* increase during pregnancy, this population remains small. The lack of overlap between *Slc5a4a*‐positive cells and *Ki67*‐positive cells further supports the idea that SGLT3a regulates progenitor proliferation through a cell‐extrinsic mechanism during reproduction. It would be interesting to determine whether *SGLT3a*‐positive enterocytes sustain *Fgfbp1*‐positive progenitor proliferation and differentiation by supplying metabolites, similar to the finding that Paneth cells support *Lgr5*‐positive CBCs by providing lactate (Rodríguez‐Colman et al. 2017).

### 3.4 Upstream Mechanisms Triggering *SGLT3a* Upregulation

What upstream signals induce *SGLT3a* expression during pregnancy? In non‐pregnant mice, SGLT3 genes—*Slc5a4a* and *Slc5a4b*—are downregulated in obesity models induced by either a high‐fat diet or leptin deficiency, suggesting that SGLT3a may be regulated by metabolic or hormonal cues associated with energy imbalance (Soták et al. 2021). Notably, *SGLT3a* upregulation in early pregnancy occurs before the substantial increase in maternal food intake that occurs in mid‐pregnancy (Ameku et al. 2025). We also observed reproductive *SGLT3a* upregulation in germ‐free mice (unpublished data), indicating that this induction is independent of increased food intake and changes in gut microbiota composition.

Reproductive hormones are likely key contributors (Figure 2a). Single‐cell transcriptomic analysis of the intestinal epithelium revealed widespread prolactin receptor expression, while estrogen and progesterone receptors were absent or expressed at very low levels (Ameku et al. 2025). Consistent with this, prolactin treatment upregulated *SGLT3a* expression in intestinal organoids (Ameku et al. 2025). However, in vivo prolactin administration—whether via osmotic pump or intraperitoneal injection in virgin females—failed to induce *SGLT3a* expression (unpublished data), suggesting that additional hormonal interactions may be required for prolactin's effect in vivo. It will be important to determine whether placenta‐ or fetus‐derived factors contribute to *SGLT3a* induction during pregnancy. Supporting this possibility, *SGLT3a* upregulation is absent in pseudopregnant mice, which are hormonally pregnant but lack placental and fetal development (unpublished data).

In addition to hormonal cues, it is also possible that RANK–RANKL signaling contributes to the induction of *SGLT3a* in the intestinal epithelium during reproduction. However, whether these pathways act cooperatively to regulate maternal gut epithelial remodeling remains unknown. They may function in a compensatory manner: SGLT3a promotes accelerated epithelial turnover during reproduction, whereas RANK–RANKL protects epithelial cells from death, together helping to maintain balanced epithelial dynamics (Figure 1).

### 3.5 Roles of SGLT3a in Intestinal Physiological Functions During Reproduction

Does SGLT3a contribute to maternal intestinal physiological adaptations? Although our data indicate that SGLT3a is required to sustain maternal villus growth during reproduction, SGLT3^KO^ mice appear physiologically normal compared to WT controls based on the following: (1) Daily food intake, body composition, and glucose tolerance do not differ between WT and KO mice regardless of reproductive status; (2) Trans‐epithelial ion transport during lactation remains unchanged in KO mice, as shown by ex vivo Ussing chamber assays; (3) Digestive efficiency is comparable between lactating WT and KO mice (Ameku et al. 2025).

Nevertheless, we found that reproductive upregulation of *SGLT3a* is most pronounced in bottom‐ and mid‐villus enterocytes (Ameku et al. 2025). Given that enterocytes exhibit position‐dependent differences in absorptive properties and metabolism along the villus axis (Moor et al. 2018; Zhang et al. 2024; Zwick et al. 2024), SGLT3a may modulate the quality or composition of nutrients absorbed by the intestine rather than overall digestive capacity. Consistent with this idea, single‐cell transcriptomics data show altered expression of metabolic pathway genes in absorptive enterocytes lacking SGLT3a, particularly genes of the solute carrier (SLC) family, including sodium‐ or proton‐coupled transporters, consistent with our electrophysiological findings (Ameku et al. 2025). These results suggest that while SGLT3a is not essential for global intestinal function, it supports metabolic plasticity—at least at the transcript level—in absorptive enterocytes during reproduction. Future studies should examine whether SGLT3a loss affects metabolic protein abundance or metabolite profiles in the intestinal epithelium in a reproduction‐dependent manner.

Maternal gut growth, including villus growth, is associated with reproductive fitness. Offspring raised by SGLT3a^KO^ mothers show significantly reduced viability by lactation day 7, despite similar litter sizes at birth between WT and KO mothers (Ameku et al. 2025). Consistent with this, Onji et al. (2025) found that impaired maternal villus growth caused by *Rank* deletion reduces milk quality, resulting in compromised offspring growth and metabolic health. Further studies are needed to investigate the impact of maternal gut remodeling on fetal development during pregnancy and on effects on pup survival and growth following multiple pregnancies.

## 4 Outlook

During reproduction, maternal organs undergo marked structural and functional adaptations, not only in the small intestine but also in other digestive organs, notably the pancreas (Baeyens et al. 2016; Bone and Taylor 1976; Green and Taylor 1972; Hellman 1960; Ruiz‐Otero et al. 2024; Van Assche 1974; Ziegler et al. 1978, 1985) and the liver (Dai et al. 2011; He et al. 2023; Hollister et al. 1987; Kozuki et al. 2022, 2023; Milona et al. 2010; Yang et al. 2025), to meet the increased metabolic and nutritional demands of pregnancy and lactation. Similar reproductive remodeling occurs in non‐digestive tissues, with proliferation and differentiation of stem and progenitor cells reported in the brain (Chaker et al. 2023; Shingo et al. 2003), mammary gland (Asselin‐Labat et al. 2010; Corral et al. 2025; Joshi et al. 2010), bone marrow (Nakada et al. 2014), kidney (Conte et al. 2025), and abdominal skin (Ichijo et al. 2017, 2021), supporting behavioral, endocrine, immune, and physical adaptations required for offspring fitness. Reproductive hormones, including prolactin, estrogen, and progesterone, are critical for sustaining this maternal organ remodeling (Ammari et al. 2023; Asselin‐Labat et al. 2010; Baeyens et al. 2016; Conte et al. 2025; Joshi et al. 2010; Nakada et al. 2014; Paolino et al. 2021; Shingo et al. 2003). These hormones coordinate physiological responses across multiple systems. Understanding inter‐organ communication will be essential to reveal how maternal organ remodeling functions as an integrated network supporting offspring development. Further work is needed to define the mechanisms—hormonal, metabolic, and microbiota‐mediated—that drive these adaptations (Figure 2a–d).

## Conflicts of Interest

The author declares no conflicts of interest.

## Data Availability

Data sharing not applicable to this article as no datasets were generated or analyzed during the current study.
